# Role of the occupational disease consultant in the multidisciplinary discussion of interstitial lung diseases

**DOI:** 10.1186/s12931-022-02257-6

**Published:** 2022-12-08

**Authors:** Ségolene Carlier, Mouhamad Nasser, Emmanuel Fort, Céline Lamouroux, Salim Si-Mohamed, Lara Chalabreysse, Jean-Michel Maury, Rémi Diesler, Vincent Cottin, Barbara Charbotel

**Affiliations:** 1 Université de Lyon, Université Claude Bernard Lyon 1, Université Gustave Eiffel-IFSTTAR, UMRESTTE, UMR T 9405, Domaine Rockefeller, 69373 Lyon Cedex 08, France; 2 Hospices Civils de Lyon, CRPPE-Lyon, Centre Hospitalier Lyon Sud, 69495 Pierre Bénite, France; 3 National Reference Center for Rare Pulmonary Diseases, Hôpital Louis Pradel, Hospices Civils de Lyon, Université de Lyon, INRAE, Lyon, France; 4 Department of Cardiovascular and Thoracic Radiology, Hôpital Louis Pradel, Hospices Civils de Lyon, 59 Boulevard Pinel, 69500 Bron, France; 5 UMR 5220, CREATIS, INSA Lyon, Université Claude Bernard, Lyon 1, Lyon, France; 6 Département de Chirurgie Thoracique, Transplantation Pulmonaire et Cardio-Pulmonaire, Hôpital Louis Pradel, Hospices Civils de Lyon, Lyon, France; 7 CICLY LYON, Centre Pour L’innovation en Cancérologie de Lyon, Lyon, France; 8 Service d’anatomie-Pathologique, Groupement Hospitalier Est, Hospices Civils de Lyon, Bron, France; 9 UMR754, INRAE, Université Claude Bernard Lyon 1, Université de Lyon, Lyon, France

**Keywords:** Interstitial lung disease, Multidisciplinary discussion, Occupational exposure, Asbestos, Silica

## Abstract

**Background:**

Diffuse interstitial lung diseases (ILD) constitute a heterogeneous group of conditions with complex etiological diagnoses requiring a multidisciplinary approach. Much is still unknown about them, particularly their relationship with occupational exposures. The primary objective of this study was to investigate the distribution of occupational exposures according to type of ILD. The secondary objectives were to estimate the proportion of ILDs possibly related to occupational exposure and to evaluate the added value of the participation of an occupational disease consultant in ILD multidisciplinary discussions (MDD).

**Methods:**

From May to December 2020, all consecutive patients with ILD whose cases were reviewed during a MDD in a referral centre for ILD were prospectively offered a consultation with an occupational disease consultant.

**Results:**

Of the 156 patients with ILD whose cases were reviewed in MDD during the study period, 141 patients attended an occupational exposure consultation. Occupational exposure was identified in 97 patients. Occupational exposure to asbestos was found in 12/31 (38.7%) patients with idiopathic pulmonary fibrosis (IPF) and in 9/18 (50.0%) patients with unclassifiable fibrosis. Occupational exposure to metal dust was found in 13/31 (41.9%) patients with IPFs and 10/18 (55.6%) patients with unclassifiable fibrosis. Silica exposure was found in 12/50 (24.0%) patients with autoimmune ILD. The link between occupational exposure and ILD was confirmed for 41 patients after the specialist occupational consultation. The occupational origin had not been considered (n = 9) or had been excluded or neglected (n = 4) by the MDD before the specialised consultation. A total of 24 (17%) patients were advised to apply for occupational disease compensation, including 22 (15.6%) following the consultation. In addition, a diagnosis different from the one proposed by the MDD was proposed for 18/141 (12.8%) patients.

**Conclusions:**

In our study, we found a high prevalence of occupational respiratory exposure with a potential causal link in patients with ILD. We suggest that a systematic specialised consultation in occupational medicine could be beneficial in the ILD diagnostic approach.

## Introduction

Interstitial lung disease (ILD) constitutes a heterogeneous group of diseases that each has a characteristic clinic-radiographic-pathologic pattern. The clinical presentation is non-specific, and the progressive onset of dyspnea and cough is often disabling.

ILDs are rare: the overall prevalence has been estimated to 60–80 cases per 100,000 persons, and the incidence to 30 cases per 100,000 person-years [1]. ILDs are classified into four groups according to the ATS (American Thoracic Society)/ERS (European Respiratory Society) classification published in 2013 [2]: (i) ILDs of known cause (drug-induced, environmental, connective tissue disease, vasculitis); (ii) idiopathic ILDs; (iii) pulmonary granulomatosis, including sarcoidosis; and (iv) rare forms of ILD, including lymphangioleiomyomatosis, Langerhans pulmonary histiocytosis, chronic eosinophilic lung disease, and pulmonary alveolar proteinosis.

An association between occupational exposure and ILD, including pneumoconiosis and hypersensitivity pneumonitis, has been frequently reported among the various possible aetiologies. For example, certain occupational exposures, notably to wood dust, metal dust, and silica, have been reported to be significantly associated with idiopathic pulmonary fibrosis [3]. It is difficult to differentiate asbestosis from idiopathic pulmonary fibrosis (IPF), as the diagnosis of asbestosis relies on the evidence of high exposure to asbestos in patients with pulmonary fibrosis [4]. Cryptogenic organising pneumonia has been described in workers exposed to a textile dye [5, 6], food flavourings [7–11], and workers in the glass fibre-reinforced plastic industry [12]. ILD cases have also been described among workers of the nylon flock industry [13]. Exposure to dust or fumes was reported in 72% of patients with desquamative interstitial pneumonia (DIP) [14]. Pulmonary fibrosis and alveolar proteinosis have been reported in workers who manufacture liquid crystal displays, and alveolar proteinosis has also been reported in workers exposed to occupational dust [15–17].

An association between scleroderma and exposure to solvents has been demonstrated in meta-analyses [18, 19], and more recently an association between scleroderma and exposure to heavy metals has also been reported, but the latter requires further investigation [20]. Two French case–control studies found a significant correlation between scleroderma and exposure to welding fumes, however, a meta-analysis including two additional case–control studies did not confirm this association [19].

The French agency for food, environmental, and occupational health safety (ANSES; Agence nationale de sécurité sanitaire de l'alimentation, de l'environnement et du travail) reported a strong and certain causal link between silica exposure and systemic scleroderma in 2019 [21]. A causal link between silica exposure and systemic lupus erythematosus and rheumatoid arthritis was also considered definite. The ANSES reported an increase in the occurrence of autoimmune diseases among workers exposed to silica and concluded to a possible causal relationship between silica exposure and ANCA (anti-neutrophilic cytoplasmic autoantibody)-associated vasculitis, but to a lack of evidence for a causal relationship between silica and Sjögren's syndrome or autoimmune myositis [21].

The etiological diagnosis of ILD is difficult as clinical, radiological, and histopathological features need to be taken into consideration [22, 23]. The essential role of multidisciplinary discussions in the diagnosis of ILD has been recognized, and it has become the gold standard for ILD diagnosis [24]. However, diagnostic uncertainties can still be present and, therefore, the concept of 'working diagnosis' is used [25]. It is essential to have information about patients’ occupational and environmental exposures for the diagnosis of ILD. Because of the complexity of the occupational interview, an occupational disease consultant should collect this information [26]. Moreover, an official ATS/ERS joint report identified the urgent need to improve knowledge about the role of occupational factors in the context of non-malignant respiratory diseases [3].

We performed a prospective study whose primary objective was to investigate the type and distribution of occupational exposures according to the types of ILD. The secondary objectives were to estimate the proportion of ILDs with an occupational cause and to evaluate the medico-social impact of the participation of an occupational disease consultant in multidisciplinary discussions (MDD) followed by a specialist consultation.

## Methods

Between May and December 2020, a specialised occupational disease consultation was systematically offered to all patients with ILD whose cases were reviewed in a MDD in an expert centre for rare lung diseases. Patients with sarcoidosis were not included as only patients with severe or refractory sarcoidosis are referred for MDD, and therefore discussed cases are not representative of the population.

The interview, which was carried out or supervised by a single experienced occupational disease consultant, consisted of two parts. During the first part, the patients’ job history was reviewed in order to assess the tasks performed and the products used during their career. Environmental exposures were also assessed. The data from these interviews were collected to create a database including the following variables: age, sex, smoking status, non-occupational risk factors, clinical data about the ILD, professional exposures, professional career coded using the ISCO-2008 (International Standard Classification of Occupations) and NAF-2008 (French Nomenclature of Activities), and existence of any previous occupational disease claims.

**Fig. 1:**
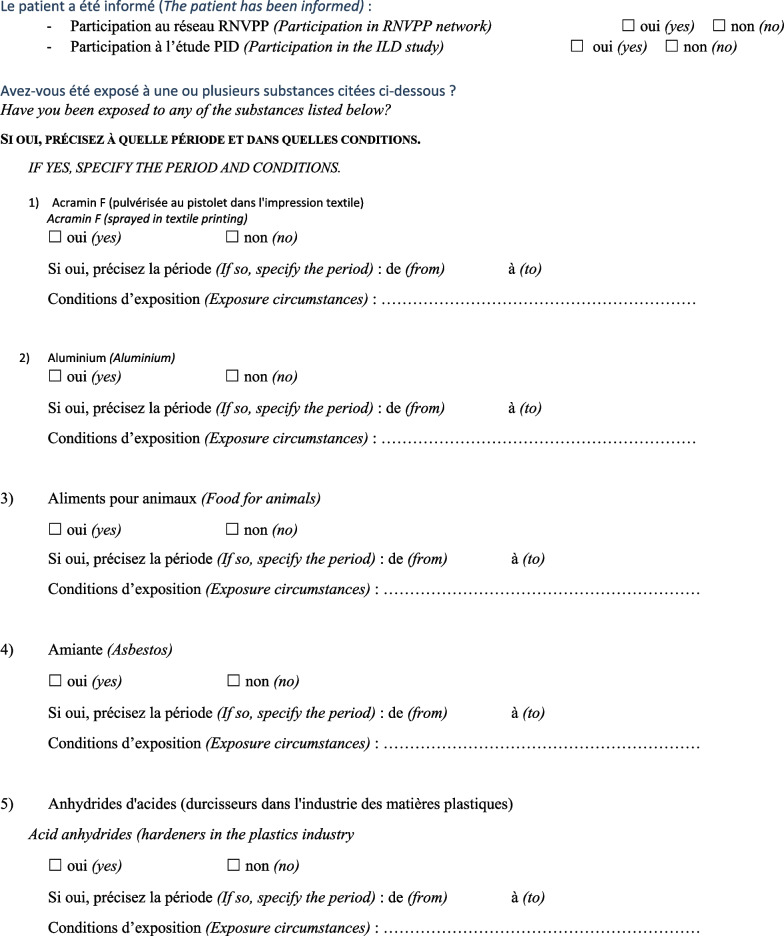

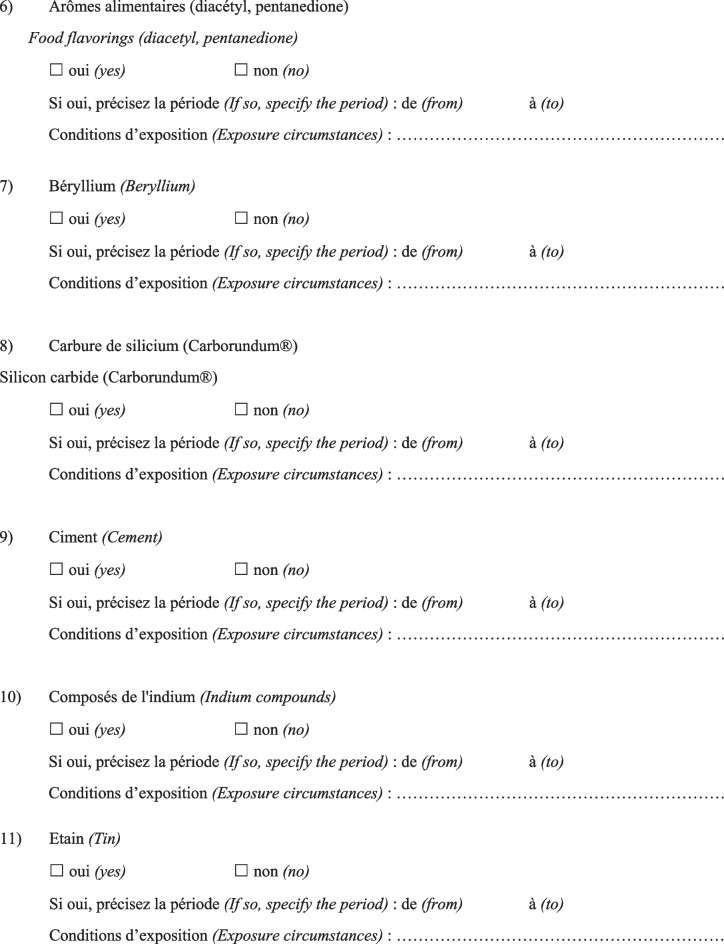

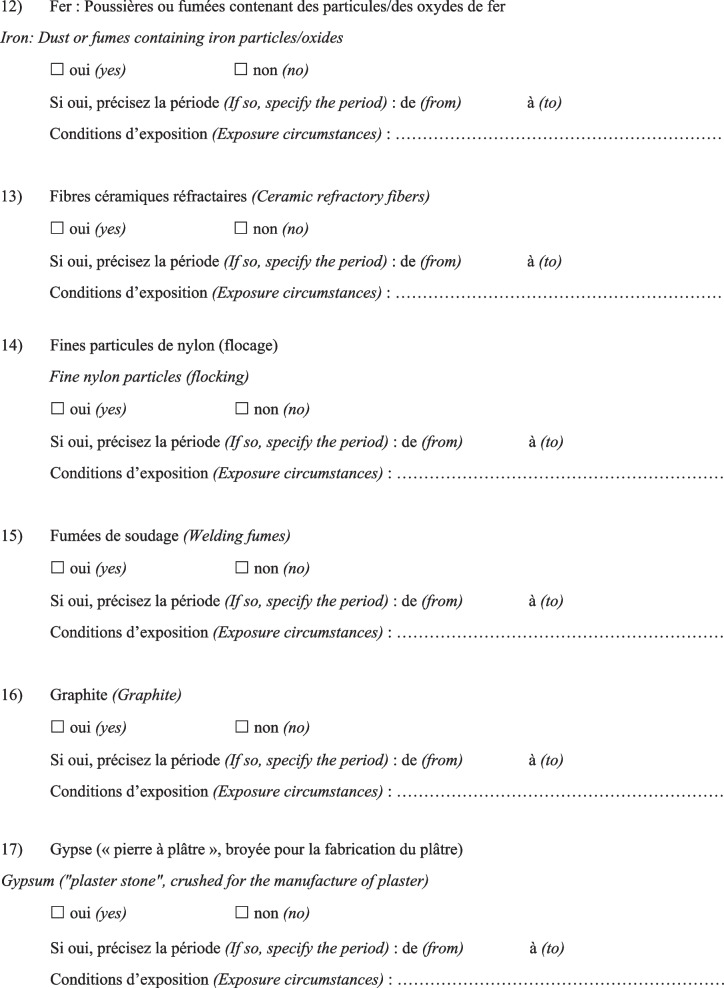

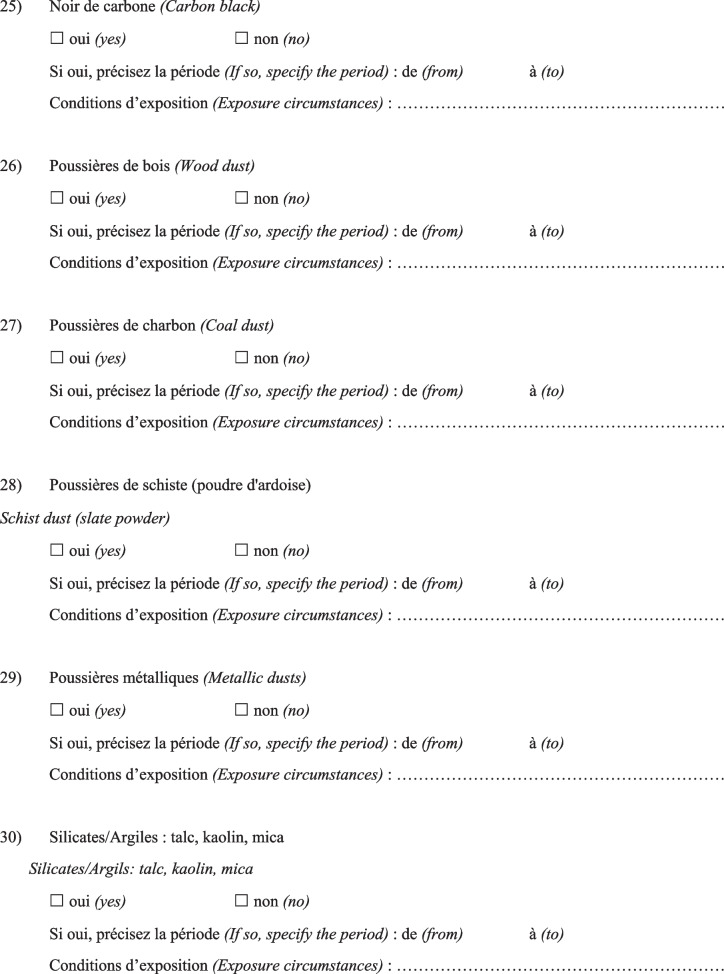

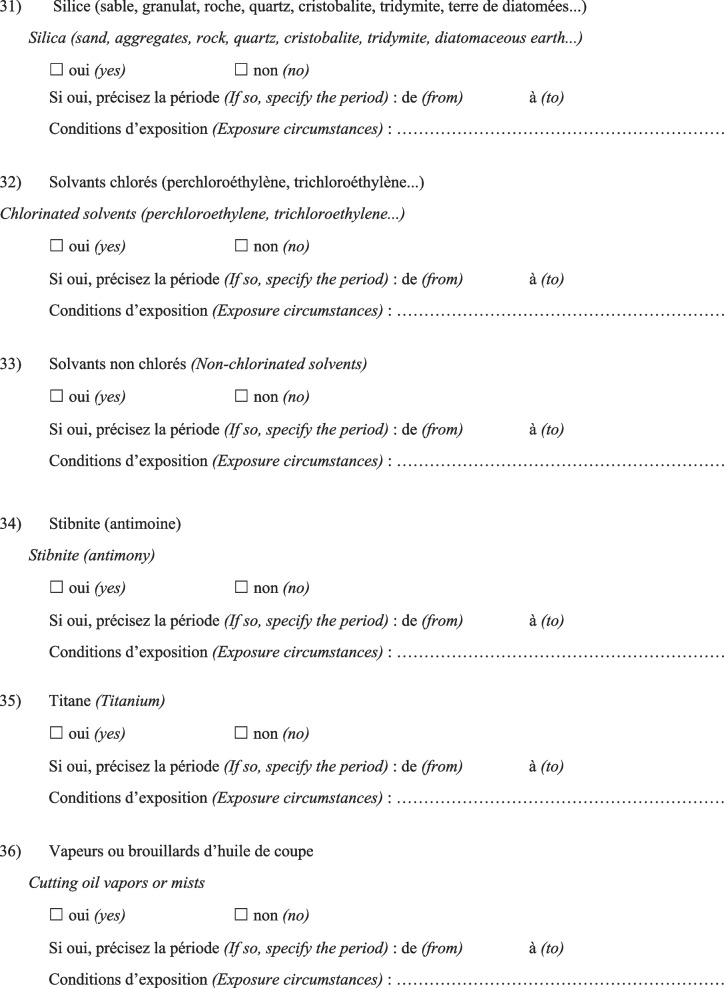

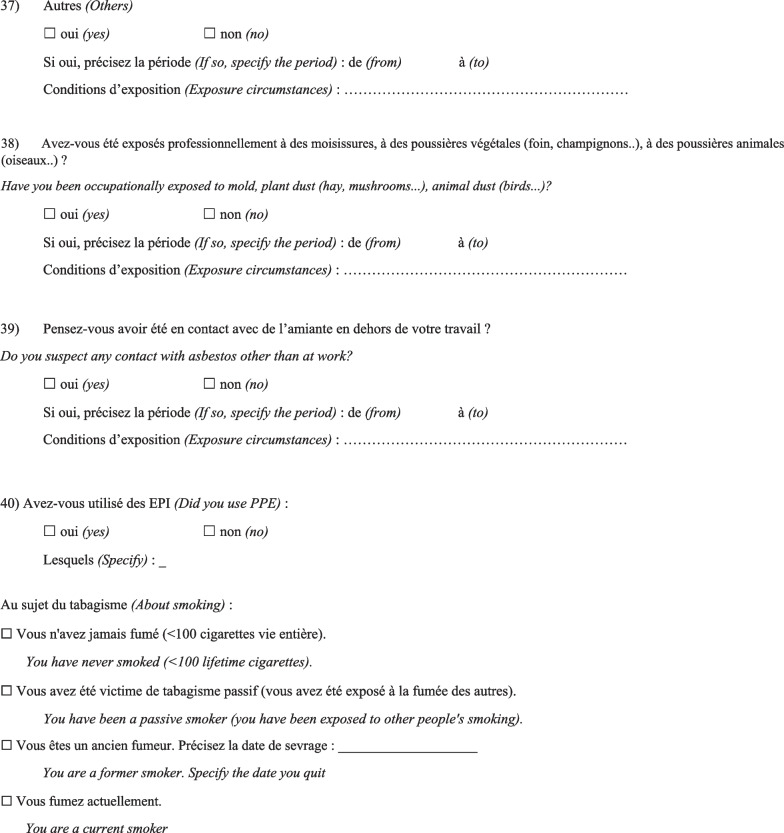

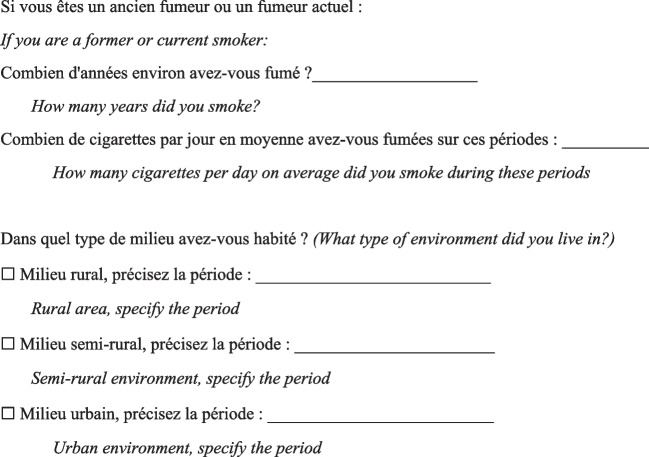
Occupational exposure inventory questionnaire for patients with ILD

Then, the physician completed a questionnaire (Fig. 1) based on a list of specific substances that had been previously identified as known or suspected fibrogenic substances from published studies. Finally, a definite, high-confidence, or conditional diagnosis of exposure-related ILD could be made (with an estimated probability of 70% or greater according to the diagnostic ontology proposed by Ryerson et al*.* [27] and previously applied to the diagnosis of pulmonary fibrosis [28]). Then, patients were advised to apply for occupational disease compensation and an initial medical certificate of occupational disease was issued. The medical impact of the specialised occupational consultation was also assessed regarding the possible suggestion of a diagnosis other than the one initially mentioned during the MDD.

Quantitative were expressed as mean (± standard deviation, SD) and qualitative variables as count (percentage). Associations between exposure and ILD were analysed using a Chi-square test or the Fisher's exact test, and p-value < 0.05 was considered significant.

The participants received written information describing the study, its objectives, and the nature of the data collected, and were informed about their right to choose to participate or not. The study protocol was approved by the scientific and ethics committee of the *Hospices Civils de Lyon* (HCL, No. 20_257 on 04/12/2020) and complied with the French data protection authority (CNIL, Commission nationale de l’informatique et des libertés) reference method MR004 and was registered under the number 21_5257 in the HCL CNIL register.

## Results

A total 156 consecutive patients were invited to attend the specialist consultation and 141 (90%) accepted: 10 refused, 4 could not be reached, and 1 patient attended the consultation but refused the use of his/her data. The mean (± SD) age of the included patients was 66 (± 12.5) years (Table 1). For active or ex- smokers, the estimated mean consumption was 22 pack-years, it was higher for men (23 pack-years) than for women (15 pack-years; p = 0.04). The diagnoses established during the multidisciplinary discussions are summarised in Table 2. For some patients, several diagnoses were retained.

**Table 1:** Socio-demographic characteristics of patients

	Count (%)
Age at the time of consultation (years)	
≤ 59	32 (22.7%)
[60–69]	43 (30.5%)
[70–79]	55 (39.0%)
≥ 80	11 (7.8%)
Mean ± standard deviation	66 ± 12.5
Sex	
Male	89 (63.1%)
Smoker status	
Non-smoker	50 (35.5%)
Past smoker	84 (59.6%)
Active smoker	7 (5.0%)
Level of education	
No diploma	38 (27.0%)
Level 3 (GCSE)	48 (34.0%)
Level 4 (A-level)	14 (9.9%)
Level 5 (Higher national diploma)	10 (7.1%)
Level 6 (BSc/BA)	13 (9.2%)
Level 7 (MSc/MA/MBA)	14 (9.9%)
Level 8 (PhD)	4 (2.8%)

**Table 2:** Occupational origin of interstitial lung diseases (ILD) based on the multidisciplinary discussion diagnosis

Interstitial lung disease (ILD)	Count (%)	Occupational origin suggested
		Count (%)
**ILD associated with autoimmune disease**	**50 (35.5)**	**10 (20.0)**
Scleroderma	18 (36.0)	4 (22.2)
Rheumatoid arthritis	8 (16.0)	3 (37.5)
Mixed connective tissue disease	5 (10.0)	1 (20.0)
Dermatomyositis	9 (18.0)	1 (11.1)
Sjögren syndrome	4 (8.0)	1 (25.0)
Other autoimmune interstitial lung disease	3 (6.0)	1 (33.3)
ANCA (anti-neutrophilic cytoplasmic autoantibody) vasculitis	7 (14.0)	0 (0.0)
*Granulomatosis with polyangiitis*	4	
*Eosinophilic granulomatosis with polyangiitis*	2	
*Unspecified ANCA vasculitis*	1	
Idiopathic pulmonary fibrosis (IPF)*	**31 (22.0)**	**14 (45.2)**
Uncertain IPF or asbestosis	2	
**Fibrosis, unclassifiable**	**18 (12.8)**	**7 (38.9)**
**Pleuro-parenchymal fibroelastosis**	**11 (7.8)**	
Idiopathic	7	0 (0.0)
Secondary	4	1 (25.0)
**Combined Pulmonary fibrosis and emphysema**	**11 (7.8)**	**4 (36.4)**
**Fibrotic hypersensitivity pneumonitis**	**9 (6.4)**	**1 (11.1)**
**Idiopathic non-specific interstitial pneumonia (NSIP)**	**5 (3.6)**	**1 (20.0)**
**Cryptogenic organizing pneumonia (COP)**	**5 (3.6)**	**0 (0.0)**
Secondary OP and infectious episodes	1	
Asbestosis**	**3 (2.1)**	**3 (100.0)**
Uncertain IPF or asbestosis	2	
**Pulmonary alveolar proteinosis**	**3 (2.1)**	**2 (66.7)**
**Silicosis**	**1 (0.7)**	**1 (100.0)**
**Drug-induced ILD**	**1 (0.7)**	
**Pulmonary Langerhans cell histiocytosis**	**1 (0.7)**	**1 (100.0)**
**Giant cell ILD**	**1 (0.7)**	
**Borderline idiopathic hypereosinophilic syndrome and eosinophilic granulomatosis with polyangiitis**	**1 (0.7)**	
**Smoking-related ILD**	**3 (2.1)**	
Smoking-related interstitial fibrosis	2	
Unspecified smoking-related interstitial fibrosis	1	

For some patients, several diagnoses were retained

Statistically significant association: *p = 0.0256; **p = 0.0233

### Occupational exposure

An occupational exposure was considered present for 97 (68.8%) patients following the specialised consultation. The main exposures were metal dusts (50 patients), silica (40 patients), non-chlorinated solvents (39 patients), and asbestos (39 patients).

### Association of occupational exposure with ILD

The occupational disease consultant considered that the occupational exposure was related to the development of ILD in 41 (29.1%) cases; among them, 28 had been previously classified as having a suspected occupational origin during the MDD prior to the specialised consultation. Two categories of diagnoses made by the MDD were statistically significantly associated with the specialist's conclusion of evidence of an occupational origin: idiopathic pulmonary fibrosis (p = 0.0256) and asbestosis (p = 0.0233; Table 2). Patients for whom the occupational origin of ILD was evidenced were more often exposed to aluminium (p = 0.0006), asbestos (p < 0.0001), cement (p = 0.0002), tin (p = 0.0033), iron (p < 0.0001), refractory ceramic fibres (RCF; p = 0.002), welding fumes (p = 0.0002), mineral wools (p < 0.0001), plastics (p = 0.0069), hard metals (p = 0.0312), wood dusts (p = 0.0002), metal dusts (p < 0.0001), silica (p < 0.0001), chlorinated solvents (p = 0.0246), and cutting oils (p = 0.0162).

### ILDs associated with occupational exposures

Among the 31 patients diagnosed with IPF, according to the MDD, 12 (38.7%) had occupational exposure to asbestos, 13 (41.9%) to metal dust, and 11 (35.5%) to silica. Among the 18 patients with unclassifiable fibrosis, 9 (50.0%) had occupational exposure to asbestos, 10 (55.6%) to metal dust, and 9 (50.0%) to silica. Among the 50 patients with ILD associated with autoimmune disease, 12 (24.0%) had an occupational exposure to silica (Table 3).

**Table 3:** Occupational exposures for the various multidisciplinary discussion diagnoses

Exposure (n)	Autoimmune ILD (n = 50)	IPF (n = 31)	Fibrosis, un-classifiable (n = 18)	PFES (n = 11)	FHP (n = 9)	Idiopathic PPF (n = 7)	COP (n = 5)	NSIP (n = 5)	Secondary PPF (n = 4)	Asbestosis (n = 3)	PAP (n = 3)	PLCH (n = 1)	Silicosis (n = 1)
Metal dust (n = 50)	12 (24.0)	13 (41.9)	10 (55.6)	3 (27.3)	2 (22.2)	2 (28.6)	1 (20.0)	2 (40.0)	2 (50.0)	3 (100)	3 (100)	0	1 (100)
Aluminium (n = 21)	3 (6.0)	7 (22.6)	3 (16.7)	2 (18.2)	1 (11.1)	2 (28.6)		0	0	3 (100)	2 (66.7)	0	0
Tin (n = 20)	5 (10.0)	6 (19.4)	5 (27.8)	2 (18.2)	0	2 (28.6)		1 (20.0)	0	0	0	0	0
Iron (n = 41)	7 (14.0)	12 (38.7)	9 (50.0)	2 (18.2)	0	3 (42.9)	1 (20.0)	1 (20.0)	2 (50.0)	2 (66.7)	3 (100)	0	1 (100)
Welding (n = 30)	7 (14.0)	10 (32.3)	6 (33.3)	2 (18.2)	0	1 (14.3)		2 (40.0)	0	1 (33.3)	2 (66.7)	0	1 (100)
Hard metals (n = 6)	3 (6.0)	2 (6.5)	1 (5.6)	0	0	0		0	0	0	0	0	1 (100)
Silica (n = 40)	12 (24.0)	11 (35.5)	9 (50.0)	2 (18.2)	0	0		0	1 (25.0)	3 (100)	2 (66.7)	1 (100)	1 (100)
Asbestos (n = 39)	9 (18.0)	12 (38.7)	9 (50.0)	1 (9.1)	0	2 (28.6)		0	2 (50.0)	3 (100)	1 (33.3)	1 (100)	1 (100)
Chlorinated solvents (n = 24)	7 (14.0)	8 (25.8)	4 (22.2)	2 (18.2)	1 (11.1)	1 (14.3)		2 (40.0)	1 (25.0)	1 (33.3)	0	0	0
Wood (n = 24)	5 (10.0)	7 (22.6)	5 (27.8)	1 (9.1)	2 (22.2)	0		0	1 (25.0)	2 (66.7)	2 (66.7)	1 (100)	0
Plastics (n = 22)	7 (14.0)	5 (16.1)	6 (33.3)	1 (9.1)	0	0		1 (20.0)	0	2 (66.7)	2 (66.7)	0	1 (100)
Mineral wool (n = 21)	5 (10.0)	10 (32.3)	2 (11.1)	1 (9.1)	0	0		0	1 (25.0)	2 (66.7)	1 (33.3)	1 (100)	0
Cement (n = 26)	6 (12.0)	8 (25.8)	7 (38.9)	1 (9.1)	0	0		0	1 (25.0)	2 (66.7)	1 (33.3)	1 (100)	0
Cutting oils (n = 17)	3 (6.0)	5 (16.1)	6 (33.3)	1 (9.1)	0	0		0	1 (25.0)	0	1 (33.3)	0	0
RCF (n = 9)	2 (4.0)	1 (3.2)	3 (16.7)	0	0	0		0	0	2 (66.7)	1 (33.3)	1 (100)	1 (100)

Data are expressed as count (percentage)

*COP* Cryptogenic organising pneumonia, *FHP* fibrotic hypersensitivity pneumonitis, *ILD* interstitial lung disease, *IPF* idiopathic pulmonary fibrosis, *NSIP* idiopathic non-specific interstitial pneumonia, *PAP* pulmonary alveolar proteinosis, *PFES* pulmonary fibrosis emphysema syndrome, *PLCH* pulmonary Langerhans cell histiocytosis, *PPF* pleuro-parenchymal fibroelastosis, *RCF* refractory ceramic fibres. For some patients, several diagnoses were retained

The MDD formulated opinions on the possibility of a link between occupational exposure and ILD, before and after the specialist consultation. After this consultation, the occupational exposure related to the ILD was confirmed for 41 patients. Before this consultation, the occupational exposure related to the ILD was suggested for 45 patients and confirmed after the consultation for 28 of them, and this link was confirmed for 13 additional patients (Table 4).

**Table 4:** Summary of association between ILD and occupational exposure before and after the occupational disease consultation

	Before occupational exposure consultation, N	After occupational exposure consultation, n/N (%)
Link investigated	90	
Occupational origin suggested	45	28/45 (62.2)
Occupational origin ruled out	45	4/45 (8.9)
Link not investigated	51	9/51 (17.6)

### Initial medical certificate

Following the occupational exposure consultation, 15 patients were given an initial medical certificate for occupational disease and the decision for 7 other patients was subject to further discussion with the pulmonologists. Among the 41 patients for whom the occupational origin had been confirmed, we estimated that 24 could claim compensation for an occupational disease, regardless of whether the initial medical certificate had been given before or after the specialist consultation. For 19 patients, the disease was listed as an occupational disease in the French system (derived from the European and the international ILO list [29, 30]), for three it was not listed, and for two the disease could be listed or unlisted depending on the exposure considered. Of note, unlisted diseases can be compensated under some specific conditions. The most frequent listed diseases were on the French occupational disease table 30A (asbestosis), followed by those on table 25 A (silicosis, Caplan-Colinet syndrome, scleroderma). For the 17 other patients for whom an occupational origin seemed plausible, compensation was not possible either because they had no insurance (self-employed) or because their disease did not correspond to a listed occupational disease.

### Proposal of an alternative diagnosis

The occupational disease consultant proposed an alternative diagnosis or provided precisions to the diagnosis for 18 (12.8%) patients (asbestosis in 13 cases). For one of these patients, two different diagnoses (asbestosis and silicosis) were suggested to the pulmonologists, in view of a significant exposure to asbestos and silica, and atypical imaging. The other diagnoses were hard metal fibrosis (n = 2, 11.1%), silico-proteinosis (n = 1, 5.6%), Caplan-Colinet (n = 1, 5.6%), IPF (n = 9, 50.0%), and unclassifiable fibrotic ILD (n = 5, 27.8%). For two patients, two different diagnoses (IPF and asbestosis) were suggested before the consultation, these were eventually classified as asbestosis following the consultation with the occupational disease consultant.

Among the six possible diagnoses of asbestosis suggested before the occupational disease consultation, the diagnoses finally retained by the multidisciplinary team were asbestosis (n = 1), IPF (n = 2), uncertain diagnosis of IPF /asbestosis (n = 2), and unclassifiable fibrotic ILD (n = 1).

## Discussion

### Distribution of occupational exposures according to the type of ILD

About two thirds of the patients with ILD had an occupational respiratory exposure, including 41 for whom the hypothesis of occupational origin was plausible and 15 for whom an occupational disease compensation procedure was initiated following the occupational disease consultation.

A recent survey including 156 patients with ILD has reported that about two thirds of the patients had some occupational exposure, which is similar to the proportion found herein [26]. The most common exposures identified in that study were metal dusts, silica, solvents, and asbestos but the exposure was less frequent than in our study, particularly for metal dusts (21/156 vs. 52/141) and for silica (6/156 vs. 47/141) [26]. In the same study, more than a third of patients exposed to metal dusts had connective tissue disease-associated ILD and a quarter had IPF; half of those exposed to silica were eventually diagnosed with IPF, a third with connective tissue disease, and about a fifth with an autoimmune ILD.

The prevalence of occupational exposure to silica in our study should be compared to that of the working population, in a time frame when the study population was of working age. On the one hand, according to the periodical SUMER® French national cross-sectional survey carried out in 2003, when the study population was of working age, the prevalence of silica exposure was estimated to be 1.5% (28.4% in our study), and the prevalence of exposure to iron oxides to be 1.0% (29.1% in our study) [31]. However, the SUMER® survey is known to underestimate occupational exposure by excluding craftsmen and mine workers, and by only taking the last week worked into consideration. On the other hand, a French case–control study investigating lung cancer in males, conducted from 2001 to 2007, has assessed the mineral wools and asbestos exposures of 1350 patients and 1912 male controls whose average age was 57.5 years [32]. The estimated cumulative exposure prevalence was 23.2% for asbestos (27.7% in our study) and 13.1% (33.3% in our study) for silica, i.e. lower than those reported in our study for both asbestos and silica, which supports a role of these exposures in the development of ILD.

We found that 38.7% of the patients who were diagnosed with IPF during the MDD had an occupational exposure to asbestos, 41.9% to metal dust, 22.6% to wood dust, and 35.5% to silica. In a recent Korean study including 78 patients with IPF, the frequency of occupational exposure, assessed by two occupational disease consultants, was 5.1% for asbestos, 26.9% for metal dust, 7.7% for wood dust, and 26.9% for silica [33]. These lower values could be explained by the fact that the authors had excluded asbestosis and silicosis from the analysis.

Among the patients with a diagnosis of ILD associated with autoimmune diseases, 24.0% had an occupational exposure to silica. The French ANSES report has concluded that there was a lack of evidence to establish a quantitative dose–response relationship between silica exposure and the presence of autoimmune ILD, but that it was possible that even a low dose could lead to an autoimmune associated ILD [21].

### Estimation of the percentage of ILDs associated with an occupational origin

In contrast with other studies, the link between ILD and an occupational origin was assessed by a specialist, who confirmed it for a third of the patients.

There are few data in published studies on the proportion of ILD related to an occupational origin. In a joint ATS/ERS report, based on 11 case–control studies, the fraction attributable to occupational vapours, gases, dust, or fumes has been estimated to 26% for IPF. The fraction attributable to metal dust was 8%, wood dust 4%, and silica 3%. In this report, which reviewed 29 published studies including 1539 patients with pulmonary alveolar proteinosis, the combined prevalence of occupational exposure was estimated to 29%. The occupational exposure in 345 patients with autoimmune alveolar proteinosis from five published studies ranged from 26 to 55%, but the link between these exposures and pulmonary alveolar proteinosis was not explored. Finally, based on 15 published studies, the percentage of patients with hypersensitivity pneumonitis linked to an occupational origin was estimated to 19% [3].

In our study, a possible occupational origin was retained for almost half of patients with IPF, but in some cases, the lack of clinical or scientific knowledge did not allow this origin to be definitively confirmed. For alveolar proteinosis, the occupational origin concerned two among the three included cases. For hypersensitivity pneumonitis, we found that the diagnosis of about one in 10 patients had a possible occupational origin.

Some patients initially diagnosed with IPF by the multidisciplinary team were eventually reclassified as having asbestosis by the occupational disease consultant due to their high exposure to asbestos. The diagnosis of asbestosis is essentially based on a significant exposure to asbestos, often prolonged, although short but intense exposures may also be responsible [4]. The threshold commonly used to suggest asbestosis is indeed 20 fibres/mL*years [34, 35].

A significant linear relationship between IPF deaths and the importation of asbestos in the United Kingdom has been reported, a relationship similar to that observed with mesothelioma deaths [36]. It has been concluded that low-level exposure to asbestos may lead to the development of IPF. In a Korean study including 1311 patients with IPF, associations between occupational exposure to dust (wood, metal, sand, stone, diesel, chemicals) and an earlier onset of the disease and excess mortality has been reported [37]. However, occupational exposure is not always related to a poor prognosis since asbestosis has a slow progression [4].

### Evaluation of the medico-social impact of the systematic addition of a specialised occupational interview

The results of our study highlighted the medico-social contribution of a specialised occupational consultation in the multidisciplinary evaluation of patients with ILD [38]. From a medico-legal point of view, this consultation resulted in 15.6% (22/141) of the patients being eligible or potentially eligible to apply for a compensation procedure. From a diagnostic point of view, the consultation resulted in a different diagnosis being proposed for 12.8% (18/141) of patients. Our findings are consistent with those of a previous study that has analysed the value of knowledge about both occupational and environmental exposure and medical history in a multidisciplinary setting, and has emphasised its contribution to the establishment of a clinical diagnosis for ILDs whose consensus diagnosis differed from the one initially proposed [39].

### Strengths and weaknesses

One of the strengths of our study lies in its relatively large sample size, and in the detailed assessment of occupational exposures by a specialist. Our case series appears to be consistent with published data regarding the repartition of ILD subtypes [40]. In addition, the occupational qualification levels of the included patients was similar to those reported by INSEE in the older general French population [41].

Even when the MDD reaches consensus diagnosis, some uncertainty may remain, inherent to the complexity of the diagnosis [42]. The joint ATS/ERS has report emphasised the risk of mistaking classical pneumoconiosis with idiopathic ILD [3]. There is, therefore, a risk of overestimating occupational exposure in patients with idiopathic ILD and, thus, of wrongly liking occupational exposure with the development of ILDs. This potential risk of error has also been reported in the American INTENSITY survey including 600 patients with ILD, in which more than half of patients had reported at least one misdiagnosis and 38% at least two misdiagnoses, prior to their current diagnosis [43].

A potential bias in the assessment of exposures could not be ruled out, because the consulting physician knew the diagnosis made by the interdisciplinary team, and their questioning could have been focused on substances potentially related to the subtype of ILD diagnosed. However, the use of a standardised questionnaire helped to minimise this bias. In addition, the assessment of job history and exposure was based on the expertise of a single senior occupational disease consultant. The evaluation could be further improved with the involvement of an industrial hygienist.

The present study did not assess the proportion of patients who eventually completed the procedure of application to recognition of occupational disease, and the number of patients who obtained some financial compensation.

## Conclusions

Our study confirmed the high prevalence of occupational exposures among patients with ILD, which warrants reinforced preventive measures to reduce occupational exposure. Further studies led in other Centres for Occupational and Environmental Pathologies are needed to confirm these findings and to compare the exposure profiles to those of a control population.

## Data Availability

The datasets used and/or analysed in the present study are available upon reasonable request to the corresponding author.

## Abbreviations

ANCA: Anti-neutrophilic cytoplasmic autoantibody
ANSES: Agence nationale de sécurité sanitaire de l'alimentation, de l'environnement et du travail
ATS: American Thoracic Society
CNIL: Commission nationale de l’informatique et des libertés
COP: Cryptogenic organizing pneumonia
CT: Computerized tomography
ERS: European Respiratory Society
FHP: Fibrotic hypersensitivity pneumonitis
HCL: Hospices Civils de Lyon
ILD: Interstitial lung disease
IPF: Idiopathic pulmonary fibrosis
ISCO-2008: International Standard Classification of Occupations-2008
NAF-2008: French Nomenclature of Activities-2008
NSIP: Non-specific interstitial pneumonia
RCF: Refractory ceramic fibres
PAP: Pulmonary alveolar proteinosis
PFES: Pulmonary fibrosis emphysema syndrome
PLCH: Pulmonary Langerhans cell histiocytosis
PPF: Pleuro-parenchymal fibroelastosis
